# Changes from 2000 to 2009 in the Prevalence of HIV-1 Containing Drug Resistance-Associated Mutations from Antiretroviral Therapy-Naive, HIV-1-Infected Patients in the United States

**DOI:** 10.1089/aid.2017.0295

**Published:** 2018-08-01

**Authors:** Lisa L. Ross, Denise Shortino, Mark S. Shaefer

**Affiliations:** ^1^ ViiV Healthcare, Research Triangle Park, North Carolina.; ^2^ PAREXEL International, Durham, North Carolina.

**Keywords:** transmitted drug resistance, HIV-1, antiretroviral-naive, surveillance mutations, antiretroviral drug resistance, NNRTI mutation transmission

## Abstract

Pre-existing HIV drug resistance can jeopardize first-line antiretroviral therapy (ART) success. Changes in the prevalence of drug resistance-associated mutations (DRMs) were analyzed from HIV-infected, ART-naive, U.S. individuals seeking ART treatment from 2000 to 2009. HIV DRM data from 3,829 ART-naive subjects were analyzed by year of sample collection using International Antiviral Society-United States (IAS-USA) and World Health Organization (WHO) “surveillance” DRM definitions; minor IAS-USA-defined DRMs were excluded. IAS-USA DRM prevalence between 2000 and 2009 was 14%, beginning with 8% in 2000 and 13% in 2009. The greatest incidence was observed in 2007 (17%). Overall, IAS-USA-defined non-nucleoside reverse transcriptase inhibitor (NNRTI) DRMs were 9.5%; nucleoside reverse transcriptase inhibitor (NRTI): 4%, and major protease inhibitor (PI): 3%. The most frequently detected IAS-USA-defined DRMs by class were NNRTI: K103N/S (4%), NRTI: M41L (1.5%), and PI: L90M (1%). Overall, WHO-defined DRM prevalence was 13% (5% in 2000; 13% in 2009). By class, NNRTI prevalence was 6%, NRTI: 6%, and PI: 3.2%. The most frequent WHO-defined DRMs were NRTI: codon T215 (3.0%), NNRTI: K103N/S (4%), and PI: L90 (1%). WHO-defined NNRTI DRMs declined significantly (*p* = .0412) from 2007 to 2009. The overall prevalence of HIV-1 containing major IAS-USA or WHO-defined DRMs to ≥2 or ≥3 classes was 2% and <1%, respectively. The prevalence of HIV-1 with WHO-defined dual- or triple-class resistance significantly declined (*p* = .0461) from 2008 (4%) to 2009 (<1%). In this U.S. cohort, the prevalence of HIV-1 DRMs increased from 2000 onward, peaked between 2005 and 2007, and then declined between 2008 and 2009; the detection of WHO-defined dual- or triple-class DRM similarly decreased from 2008 to 2009.

## Introduction

HIV-1 drug resistance may be acquired in response to antiretroviral (ARV) drug pressure and as the prevalence of resistance mutations (drug resistance-associated mutations [DRMs]) among treatment-experienced, HIV-infected individuals has increased, so has the probability of transmission of drug resistance (TDR).^1^ Numerous factors, including viral replication fitness and transmission fitness and individual risk behavior, can influence both the prevalence and the specific types of HIV-1 drug resistance that may be observed in therapy-naive individuals.^2–6^ New combination antiretroviral treatment (cART) regimens have reduced morbidity and mortality for individuals infected with HIV-1.^7^ In the United States (USA), access to cART has increased over time.

The chances that an HIV-1-infected patient has acquired virus containing TDR are often dependent on how prevalent these mutations are in the population of HIV-infected persons engaging in high-risk behaviors within that community. Based on the findings from the World Health Organization (WHO), the U.S. Department of Health and Human Services (DHHS) has estimated that in the USA, some European countries, Australia, and Japan, the risk of transmitted HIV-1 resistance to at least one ARV drug is in the range of 10%–17%, and the results from additional investigators suggest that up to 8% of the transmitted virus will exhibit resistance to drugs from more than one class.^7–11^

It has been postulated that if these levels continue to increase, it could compromise the effectiveness of some therapeutic regimens, and since transmitted non-nucleoside reverse transcriptase inhibitor (NNRTI) mutations, especially K103N/S, can persist for years in the absence of drug selection pressure, these NNRTI mutations are of special concern.^12^ It has been theorized that infection with TDR containing HIV-1 establishes those drug resistance-associated variants within the patient's viral reservoirs, making it less likely for wild-type HIV-1 to emerge in the absence of drug selection pressure and ultimately making treatment more difficult in those patients.^8–11^

Understanding the prevalence of TDR may also be complicated by the type of guideline used to assess resistance. The International Antiviral Society USA (IAS-USA) has created a globally recognized and widely utilized list of DRMs that can impact treatment response to specific drugs or drug classes that are observed on therapy failure.^13^ However, certain DRMs are not stable in the absence of drug selection pressure; these may revert to an intermediate form from which the more drug-resistant amino acid may be quickly selected once drug selection pressure is applied. The WHO has also generated a list of surveillance mutations for estimating TDR.^14^ There are differences between the two guidelines but both provide insight into understanding the prevalence of TDR containing HIV-1 in specific populations or regions.

To better understand the changes over time in the prevalence of TDR as well as to characterize changes over time in specific class of drugs or subtypes or of specific major DRMs and changes over time in TDR geographic distribution within the USA, the PREPARE analysis (Prevalence by REgion of Pre-existing Antiretroviral drug Resistance) analyzed the number and types of HIV-1 DRMs detected by the year of sample collection from HIV-1 ART-naive individuals enrolling into clinical trials from 2000 to 2009.

## Methods

### Data sources

All HIV-1 reverse transcriptase (RT) and protease (PRO) mutation data and demographic factors were obtained from previously completed clinical studies with study sites located in the USA. The list of 17 studies included in this analysis and the total number of subjects with HIV-1 mutation data from each study are provided in Supplementary Table S1 (Supplementary Data are available online at www.liebertpub.com/aid).

For studies that included sites outside the USA, only DRM data from U.S.-based subjects were analyzed. Within each of these clinical studies, written informed consent had previously been obtained from each subject for study procedures, including HIV-1 genotypic analysis, and each study had been approved by the ethics review boards at the participating centers and conducted in accordance with good clinical practice; see primary publications for each study for additional details.^15–31^ The HIV-1 RT and PRO mutation data had been obtained from plasma-derived HIV-1 samples collected at screening or baseline from ART-naive, HIV-infected patients between the years 2000 and 2009. The earliest available mutation data were used if more than one pretherapy result was available for a subject.

No single study supplied HIV-1 mutation data that comprised more than 15% of the analysis population. For two early studies (APV30001 and APV30002), a randomly selected subset of study subjects had pretherapy genotyping performed rather than the entire study population. Baseline demographic data from subjects whose HIV-1 genotypes were assessed in this analysis were also summarized.

### Statistical analyses

The mutation data were provided for this analysis in the form of amino acid change from wild type. The laboratories used differed by study and included Monogram Biosciences (South San Francisco, CA), VIRCO NV (Mechelen, Belgium), and Research Think Tank (Atlanta, GA). Compilation and analysis of the demographic and the HIV-1 DRM data were performed at PAREXEL International (Durham, NC).

DRMs were defined and analyzed as per IAS-USA and by WHO guidelines.^13,14^ Changes in HIV-1 overall DRM prevalence were analyzed by year of sample collection, by specific DRMs, and by geographic region. If available, subtype was also assessed for these samples. No protease inhibitor (PI) data are presented for the year 2000 as ≤20 subjects had PRO mutation data. Minor IAS-USA NNRTI and PI DRMs were excluded from the tabulation. The geographic distribution by region was as defined by the U.S. Census Bureau.^32^

The HIV-1 RT and PRO mutation and demographic data tabulations and statistical analyses were performed using SAS (SAS Institute Inc., Cary, NC) at PAREXEL International.

## Results

### Analysis population

HIV-1 RT and PRO mutation data were available for 3,829 ART-naive HIV-infected subjects collected between the years 2000 through 2009 and encompassed 36 states in the USA and the District of Columbia. The demographics of the overall population included in this analysis are shown in Table 1. The subjects were primarily male (83%) and had asymptomatic HIV infection (73%); the primary risk factor was homosexual sexual contact (65%). Almost half (49%) listed their race as white.

**Table 1.** Cohort Demographics (Total *n* = 3,829 Subjects; *n* Shown Below if Different)

Median age (range)	37 years (18–79)
Gender, *n* (%)	
Male	3,170 (83)
Female	659 (17)
Median HIV-1 RNA, *n* = 3,824 (range)	4.875 log_10_ copies/mL (1.69–7.41)
Median CD4, *n* = 3,820 (range)	231 cells/mm^3^ (1–1,179)
Centers for Disease Control Classification (*n* = 3,572), *n* (%)	
Asymptomatic HIV infection (Class A)	2,621 (73)
Symptomatic (non-AIDS) conditions (Class B)	567 (16)
Acquired immunodeficiency syndrome (Class C)	384 (11)
Race (*n* = 3,761), *n* (%)	
White	1,842 (49)
Black	1,391 (37)
Hispanic	300 (8)
Other	228 (6)
HIV-1 RNA ≤100,000 copies/mL (*n* = 3,824), *n* (%)	2,205 (58)
Risk factors (*n* = 3,108), *n* (%)	
Homosexual	2,008 (65)
Heterosexual	1,107 (36)
Injectable drug use (intravenous)	158 (5)
Transfusion	47 (2)
Occupational	23 (<1)
Other	72 (2)
Hepatitis B+ (*n* = 3,408), *n* (%)	104 (3)
Hepatitis C+ (*n* = 3,401), *n* (%)	264 (8)

HIV-1 subtype was also available for many of these samples (3,105/3,829), the majority (97%) were subtype B. The non-subtype B sequences included subtype C: 1.3%, G: 0.55%, A: 0.45%, D: 0.1%, F: 0.03% and chimeric mixes, including B/A (0.06%), B/D (0.16%), B/F (0.6%), B/G (0.1%), or A/G (0.6%).

The distribution of genotypic data by year of sample collection was as follows: 2000 (*n* = 251); 2001 (*n* = 411); 2002 (*n* = 206); 2003 (*n* = 405); 2004 (*n* = 654); 2005 (*n* = 691); 2006 (*n* = 366); 2007 (*n* = 558); 2008 (*n* = 97); and 2009 (*n* = 190).

### Summary of overall DRMs with TDR and TDR by drug class as assessed by IAS-USA or WHO definitions

From 2000 to 2009, 14% [551/3,829; 95% confidence interval (CI; 13.3%–15.5%)] (Table 2) of ART-naive HIV-infected subjects were infected with HIV-1 containing nucleoside reverse transcriptase inhibitor (NRTI), NNRTI, or major PI DRMs as defined by IAS-USA guidelines. Mutations classified as minor PI or NNRTI in the IAS-USA guidelines were not considered indicative of TDR and were not quantified for this analysis, since these polymorphisms occur in the absence of drug selection pressure and by themselves have little or no impact on drug susceptibility. The proportion of subjects whose virus contained major IAS-USA resistance mutations to two or more classes was rare (2%), 95% CI (1.5%–2.4%), and the proportion of subjects whose virus with triple class drug resistance was <1%, 95% CI (0.2%–0.6%).

**Table 2.** Summary of Drug Resistance-Associated Mutations by Drug Class Categories Using International Antiviral Society-United States or World Health Organization Surveillance Definitions

*Category*^a^	*No. of samples with HIV-1 mutations (percentage)*^b^
Major IAS-USA mutations by class	
0 Class	3,278 (86)
1 Class	476 (12)
2 Classes	59 (2)
3 Classes	16 (<1)
1, 2 or 3 Classes	551 (14)
2 or 3 Classes	75 (2)
3 Classes	16 (<1)
WHO surveillance mutations by class	
0 Class	3,346 (87)
1 Class	404 (11)
2 Classes	64 (2)
3 Classes	15 (<1)
1, 2 or 3 Classes	483 (13)
2 or 3 Classes	79 (2)
3 Classes	15 (<1)

^a^ Defined as an NRTI, NNRTI, or PI drug class; minor NNRTI and PI mutations excluded in the IAS-USA analysis.

^b^ For a small proportion (254/3,829 samples), DRMs had been obtained from partial genotype (249-sample RT data only, 5 protease data only).

DRMs, drug resistance-associated mutations; IAS-USA, International Antiviral Society-United States; NNRTI, non-nucleoside reverse transcriptase inhibitor; NRTI, nucleoside reverse transcriptase inhibitor; PI, protease inhibitor; RT, reverse transcriptase; WHO, World Health Organization.

The viral load was available for almost all subjects whose HIV-1 contained DRMs (550 of 551 subjects) and the median HIV-RNA was 4.88 log_10_copies/mL (range: 1.69–6.50 log_10_copies/mL), which was similar to the median viral load (4.88 log_10_copies/mL) observed for the overall population.

By WHO guidelines, 483/3,829 [13%, 95% CI (11.6%–13.7%)] of ART-naive subjects had HIV-1 with any surveillance resistance mutations. The proportion of subjects whose virus contained WHO-defined resistance mutations to two or more drug classes was rare [2%, 95% CI (1.6%–2.5%)], and the proportion with triple class drug resistance was <1% [95% CI (0.2%–0.6%)] (Table 2). The prevalence of these mutations was also evaluated over time by year of collection (data not presented). There was a significant decline in the prevalence of these surveillance mutations when analyzed by year of collection in the incidence of subjects with HIV-1 with WHO-defined dual- or triple-class resistance (*p* = .0461; Fisher's exact), the incidence declined from 4% in 2008 to <1% in 2009.

### The prevalence of HIV-1 containing DRMs by year and stratified by geographic region

**FIG. 1.**
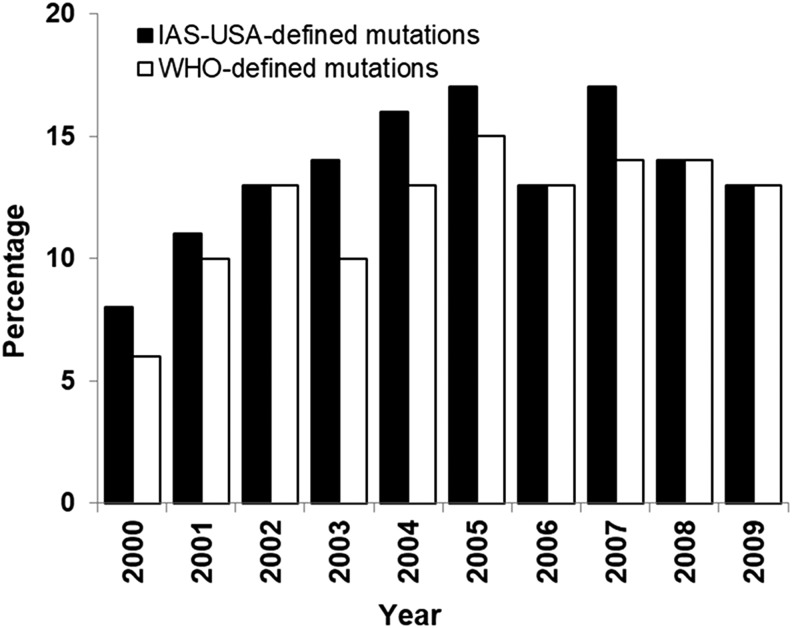
Prevalence of DRMs in ART-naive subjects from 2000 to 2009 by IAS-USA or WHO guidelines. DRMs, drug resistance-associated mutations; IAS-USA, International Antiviral Society-United States; WHO, World Health Organization.

The prevalence of HIV-1 with TDR as defined by the IAS-USA guidelines or any WHO-defined DRM was assessed by calendar year of sample collection. As seen in Figure 1, the prevalence tended to increase over time from 2000 through 2007 and to decline in 2008 and 2009.

**FIG. 2.**
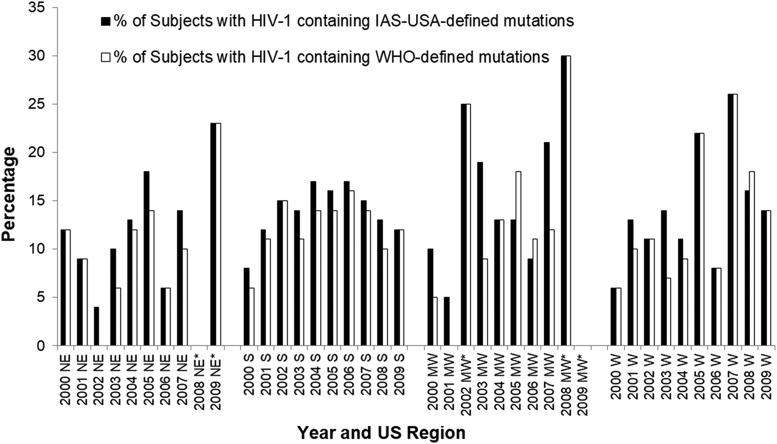
Overall yearly incidence of HIV-1 DRMs by U.S. geographic regions. Those regions where the sample numbers for that year were small (<20) are noted with an *asterisk* (*).

The prevalence of these mutations was also determined by calendar year of sample collection and stratified by geographic region as either the Southern, Northeastern, Midwestern, or Western regions. As seen in Figure 2, the DRM prevalence within a calendar year and region was generally similar when assessed by either the IAS-USA or the WHO definitions. Since 2000, the prevalence of these mutations in all geographic regions appears to have initially increased over time through 2006 or 2007.

### Summary of the prevalence of specific IAS-USA-defined mutations and by drug class

**FIG. 3.**
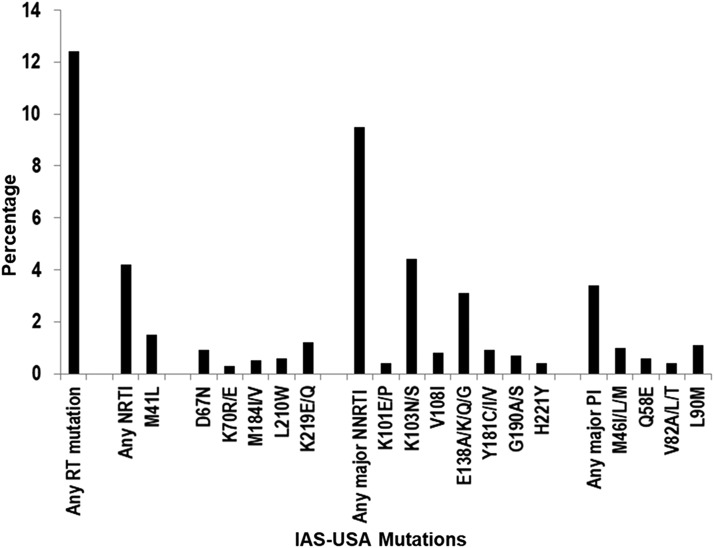
Prevalence of HIV-1 with specific IAS-USA-defined NRTI and major IAS-USA NNRTI and PI DRMs from 2000 to 2009. Only IAS-USA-defined DRMs detected in >10 viral isolates are shown. NNRTI, non-nucleoside reverse transcriptase inhibitor; PI, protease inhibitor.

The prevalence of HIV-1 by specific drug class, and by mutations within each class for the NRTI and major IAS-USA NNRTI and PI mutations was also evaluated for all samples collected from 2000 to 2009. As shown in Figure 3, the prevalence of any NRTI mutation was 4%, 95% CI: 3.5%–4.8%. The most prevalent NRTI mutation was M41L with 1.5%, 95% CI: 1.1%–1.8%. The prevalence of major NNRTI mutations was 9.5%, 95% CI: 8.5%–10.4%. The most prevalent NNRTI mutation was K103N/S with 4%, 95% CI: 3.7%–5.0%. The prevalence of major PI mutations was 3%, 95% CI: 2.8%–4.0%, while the most prevalent PI mutation was L90M [1%, 95% CI (0.8%–1.5%)]. The prevalence of IAS-USA-defined NRTI and major NNRTI and PI mutations by drug classes was also evaluated after stratification by year of collection (data not shown); no statistically significant change in prevalence by drug class by year was observed.

### Summary of the prevalence of specific WHO-defined surveillance mutations and by drug class

**FIG. 4.**
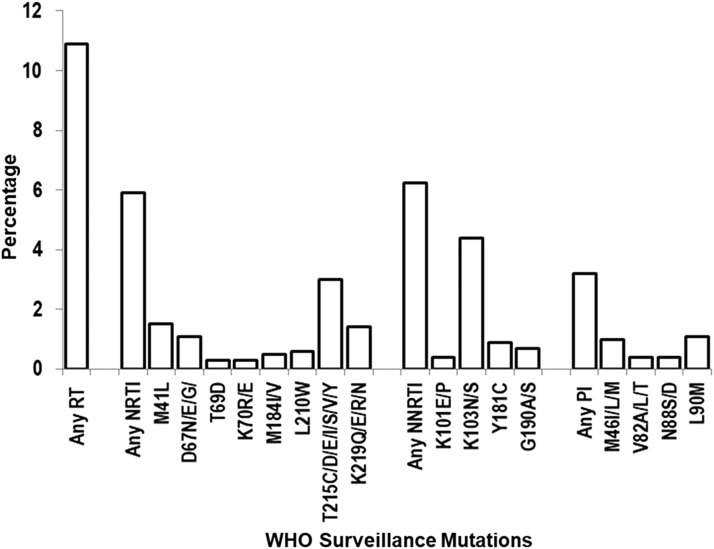
Prevalence of HIV-1 with specific WHO-defined drug resistance mutations from 2000 to 2009. Only WHO-defined surveillance mutations detected in >10 viral isolates are shown.

The prevalence of HIV-1 with specific WHO-defined DRMs was also evaluated over the collection period from year 2000 to 2009. As shown in Figure 4, the prevalence of any NRTI mutation was 6% [95% CI (5.1%–6.6%)]. NRTI mutations at codon T215, defined as either C/D/E/F/I/S/V/Y (or mixtures of these mutations), were the most frequently detected [3%, 95% CI (2.6%–3.7%)]. The prevalence of NNRTI mutations was 6% [95% CI (5.5%–7.0%)], and the most prevalent NNRTI mutation was K103N/S [4%, 95% CI (3.7%–5.0%)]. The prevalence of major PI mutations was 3% [95% CI (2.6%–3.8%)], and the most prevalent PI mutation was L90M [1%, 95% CI (0.7%–1.4%)].

**FIG. 5.**
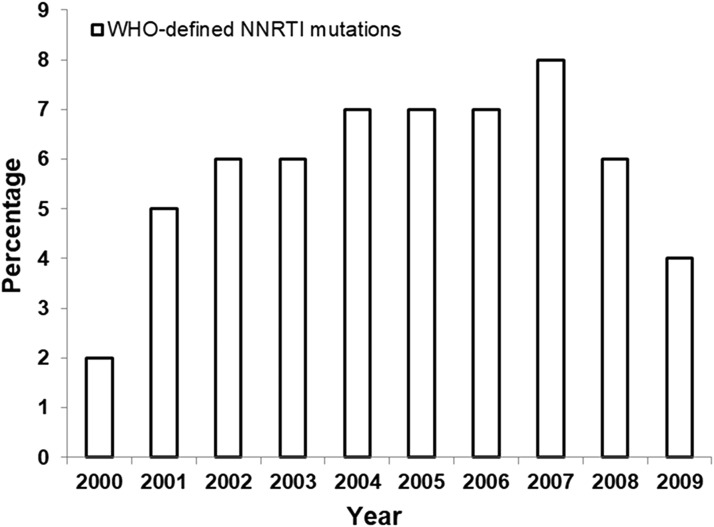
Yearly prevalence of HIV-1 with any WHO-defined NNRTI DRM in HIV-1 from ART-naive U.S. subjects from 2000 to 2009.

The prevalence of WHO-defined mutations for all drug classes was also evaluated after stratification by year of collection. The most striking change was in the DRM prevalence within the NNRTI drug class (Fig. 5). The prevalence of WHO-defined mutations for the NNRTI drug class declined significantly (*p* = .0412 by logistic regression) from 8% in 2007 to 6% in 2008 and 4% in 2009.

### Prevalence of specific NNRTI mutations in HIV-1 from ART-naive U.S. subjects stratified by year of collection

**FIG. 6.**
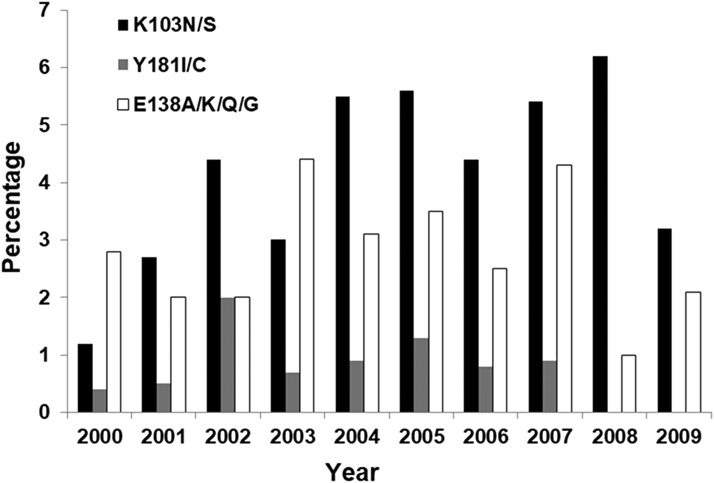
Yearly prevalence of specific HIV-1 NNRTI drug resistance mutations in HIV-1 from ART-naive subjects from 2000 to 2009.

Analysis of the three most prevalent NNRTI mutations is shown in Figure 6; the prevalence of the K103N/S mutation continued to increase until 2008 (6% in 2008) and then declined in prevalence in 2009 to 3%. For two other major NNRTI mutations, mutations at codons Y181 were not detected after 2007 and the prevalence of mutations at E138 also appeared to decline after 2007, from 4% in 2007 to 2% in 2009.

## Discussion

The prevalence of TDR remains a source of concern. Based on the results of this analysis, within the USA, the overall TDR prevalence began to rise starting with the first analysis in 2000 but by 2008 and 2009 appears to be declining, irrespective of whether TDR was calculated using IAS-USA or WHO definitions.

The drug class with the most persistent mutations, the NNRTI DRMs, appears to have declined. The time trends observed for our cohort are similar to what has been reported in a literature-based review spanning a similar period of time that examined temporal changes in the epidemiology of transmission of drug-resistant HIV-1 across various regions of the world.^33^ However, there remains a need for active surveillance to guide cART usage and to better understand the various risk group contributions to HIV transmission and resistance, which can contribute to individual treatment strategies and population-based interventional strategies.

Since 2000, when the first samples were collected for this analysis, changes in the recommendations regarding when ART could be initiated have allowed patients to begin cART treatment immediately after HIV-1 diagnosis, rather than after a decline in CD4 cell count. In addition to changes in cART access, the availability of newer ARVs for the HIV-infected U.S. population has increased the options for constructing a durable treatment regimen, and pretherapy HIV genotypic analysis is now recommended by the DHHS, which can assist clinicians to select the most effective regimen for their patients and help to prevent virological failure and accumulation of further HIV-1 resistance mutations. All of these changes likely contributed to the decline in TDR seen in this cohort by 2008 and 2009.

Despite the promising results from this analysis, suggesting that overall prevalence of TDR in the USA is declining, data from another recent study suggest that there may still be certain geographic areas within the USA where the risk of infection with HIV-1 containing TDR remains high and of serious concern.^34^ Based on the comparison of DRMs as assessed by IAS-USA versus WHO guidelines when evaluated by geographic region, no overall striking difference in TDR was observed in our analysis, suggesting that although the WHO-defined guideline is more widely utilized in this context, either guideline could have utility in detecting TDR “hotspots.” Rapid identification of “hotspots” of TDR could also enable enhanced public health follow-up of the subpopulation affected by the outbreak.

In a similar type of analysis, Margot *et al.* recently evaluated transmitted resistance utilizing data from of HIV-infected, ART-naive patients from the USA, Europe, and Asia from other previously published clinical studies.^35^ They observed an increase in prevalence for all mutations from 2000 to 2013, while a trend analysis for U.S. patients observed an increase in the any mutation and NNRTI mutation categories (both *p* < .1). The overall results are generally in agreement with what was observed in this study as the overall levels of TDR increased over time in both analyses from the levels observed in 2000, driven largely by an increase in NNRTI mutations. The latter analysis also saw a decline in the overall and NNRTI mutation prevalence, although in that cohort the decline occurred between 2010 and 2011, while in the current analysis a statistically significant decline (*p* < .05) in the prevalence of WHO-defined NNRTI mutations was observed after 2007.

The reasons for the observed differences are likely multifactorial, but given that TDR remains high within certain geographic regions, differences in the proportions of sequences obtained from specific geographic regions during the 2008 and 2009 period could have been a contributing factor. The CD4 cell counts in the latter analysis were higher than those observed in the current analysis. The lower CD4 cell counts observed in this analysis suggest that the study recruitment or study inclusion criteria permitted a higher proportion of HIV-infected patients with more established chronic HIV infections to enroll, and outgrowth of wild-type virus could have occurred in this population, leading to lower apparent prevalence of certain mutations, especially those that confer a cost to viral fitness.

In conclusion, the prevalence of transmitted drug resistance to NRTI, NNRTI, and PI as observed by population genotyping in this large cohort of HIV-1-infected, ART-naive U.S. subjects appeared to be increasing beginning in 2000 and then on the decline by 2008–2009. Notably, the presence of major NNRTI resistance WHO-defined DRM declined significantly from 2007 to 2009, while the prevalence of specific key NNRTI mutations such as K103N and Y181I/C/V also declined in 2009 compared with the preceding years. Detection of double or triple class resistance in HIV-infected antiviral-naive patients also declined significantly for the WHO-defined surveillance mutations from 2008 to 2009.

## Supplementary Material

Supplemental data
