# Supplementary material for: Changes from 2000 to 2009 in the Prevalence of HIV-1 Containing Drug Resistance-Associated Mutations from Antiretroviral Therapy-Naive, HIV-1-Infected Patients in the United States
Source: AIDS Res Hum Retroviruses. 2018 Aug 1;34(8):672–9. doi: 10.1089/aid.2017.0295 (PMC6080107; doi:10.1089/aid.2017.0295)
Supplement: Supplemental data [file Supp_Data.pdf]

Supplementary Data

Supplementary Table S1. Total Number of HIV-1 Resistance Profiles Obtained and Analyzed Before Initiating Antiretroviral Therapy from Subjects in the United States Enrolling into Clinical Studies (N=3,829; Data from Subjects Enrolled at Non-U. S. Sites Were Excluded)

Study identifier No.	*Total,* n *(%)*	*Primary study publication*
APV30001	87 (2)	Rodriguez-French *et al.*[S1]
APV30002	72 (2)	Gathe *et al.*[S2]
CNA30021	424 (11)	Moyle *et al.*[S3]
COL40263	120 (3)	Elion *et al.*[S4]
EPV20001	248 (6)	DeJesus *et al.*[S5]
ESS100327	248 (6)	Kumar *et al.*[S6]
ESS100732	453 (12)	Eron *et al.*[S7]
ESS30009	322 (8)	Gallant *et al.*[S8]
CCR100136	95 (2)	Yeni *et al.*[S9]
CCR102881	132 (3)	Currier *et al.*[S10]
COL100758	111 (3)	Hicks *et al.*[S11]
COL103952	105 (3)	Smith *et al.*[S12]
EPZ104057	567 (15)	Smith *et al.*[S13]
EPZ108859	558 (15)	Squires *et al.*[S14]
COL110408	143 (4)	Kumar *et al.*[S15]
COL111429	39 (1)	Young *et al.*[S16]
ING112276	105 (3)	Stellbrink *et al.*[S17]

Supplementary References

S1. Rodriguez-French A, Boghossian J, Gray GE, *et al.*: The NEAT study: A 48-week open-label study to compare the antiviral efficacy and safety of GW433908 versus nelfinavir in antiretroviral therapy-naive HIV-1-infected patients. J Acquir Immune Defic Syndr 2004;35:22–32.

S2. Gathe JC, Jr., Ive P, Wood R, *et al.*: SOLO: 48-Week efficacy and safety comparison of once-daily fosamprenavir/ritonavir versus twice-daily nelfinavir in naive HIV-1-infected patients. AIDS 2004;18:1529–1537.

S3. Moyle GJ, DeJesus E, Cahn P, *et al.*: Abacavir once or twice daily combined with once-daily lamivudine and efavirenz for the treatment of antiretroviral-naive HIV-infected adults: Results of the Ziagen once daily in Antiretroviral Combination Study. J Acquir Immune Defic Syndr 2005;38:417–425.

S4. Elion R, Cohen C, DeJesus E, *et al.*: Once-daily abacavir/lamivudine/zidovudine plus tenofovir for the treatment of HIV-1 infection in antiretroviral-naïve subjects: A 48-week pilot study. HIV Clin Trials 2006;7:324–333.

S5. DeJesus E, McCarty D, Farthing CF, *et al.*: Once-daily versus twice-daily lamivudine, in combination with zidovudine and efavirenz, for the treatment of antiretroviral-naive adults with HIV infection: A randomized equivalence trial. Clin Infect Dis 2004;39:411–418.

S6. Kumar PN, Salvato P, LaMarca A, *et al.*: A randomized, controlled trial of initial anti-retroviral therapy with abacavir/lamivudine/zidovudine twice-daily compared to atazanavir once-daily with lamivudine/zidovudine twice-daily in HIV-infected patients over 48 weeks (ESS100327, the ACTION Study). AIDS Res Ther 2009;6:3.

S7. Eron J, Jr., Yeni P, Gathe J, Jr., *et al.*: The KLEAN study of fosamprenavir-ritonavir versus lopinavir-ritonavir, each in combination with abacavir-lamivudine, for initial treatment of HIV infection over 48 weeks: A randomised non-inferiority trial. Lancet 2006;368:476–482.

S8. Gallant JE, Rodriguez AE, Weinberg WG, *et al.*: Early virologic nonresponse to tenofovir, abacavir, and lamivudine in HIV-infected antiretroviral-naive subjects. J Infect Dis 2005;192:1921–1930.

S9. Yeni P, LaMarca A, Berger D, *et al.*: Antiviral activity and safety of aplaviroc, a CCR5 antagonist, in combination with lopinavir/ritonavir in HIV-infected, therapy-naive patients: Results of the EPIC study (CCR100136). HIV Med 2009;10:116–124.

S10. Currier J, Lazzarin A, Sloan L, *et al.*: Antiviral activity and safety of aplaviroc with lamivudine/zidovudine in HIV-infected, therapy-naive patients: The ASCENT (CCR102881) study. Antivir Ther 2008;13:297–306.

S11. Hicks CB, DeJesus E, Sloan LM, *et al.*: Comparison of once-daily fosamprenavir boosted with either 100 or 200 mg of ritonavir, in combination with abacavir/lamivudine: 96-week results from COL100758. AIDS Res Hum Retroviruses 2009;25:395–403.

S12. Smith KY, Weinberg WG, DeJesus E, *et al.*: Fosamprenavir or atazanavir once daily boosted with ritonavir 100 mg, plus tenofovir/emtricitabine, for the initial treatment of HIV infection: 48-week results of ALERT. AIDS Res Ther 2008;5:5.

S13. Smith KY, Patel P, Fine D, *et al.*: Randomized, double-blind, placebo-matched, multicenter trial of abacavir/lamivudine or tenofovir/emtricitabine with lopinavir/ritonavir for initial HIV treatment. AIDS 2009;23:1547–1556.

S14. Squires KE, Young B, Dejesus E, *et al.*: Similar efficacy and tolerability of atazanavir compared with atazanavir/ritonavir, each with abacavir/lamivudine after initial suppression with abacavir/lamivudine plus ritonavir-boosted atazanavir in HIV-infected patients. AIDS 2010;24:2019–2027.

S15. Kumar P, DeJesus E, Huhn G, *et al.*: Evaluation of cardiovascular biomarkers in a randomized trial of fosamprenavir/ritonavir vs. efavirenz with abacavir/lamivudine in underrepresented, antiretroviral-naïve, HIV-infected patients (SUPPORT): 96-week results. BMC Infect Dis 2013;13:269.

S16. Young B, Vanig T, Dejesus E, *et al.*: A pilot study of abacavir/lamivudine and raltegravir in antiretroviral-naïve HIV-1-infected patients: 48-Week results of the SHIELD trial. HIV Clin Trials 2010;11:260–269.

S17. Stellbrink HJ, Reynes J, Lazzarin A, *et al.*: Dolutegravir in antiretroviral-naive adults with HIV-1: 96-Week results from a randomized dose-ranging study. AIDS 2013;27:1771–1778.
